# Diagnostic challenge in veterinary pathology: Does negative Ziehl-Neelsen staining rule out mycobacteriosis?

**DOI:** 10.1177/03009858251331342

**Published:** 2025-04-02

**Authors:** Anna Huupponen, Sini Ulmanen, Thomas Grönthal, Taru Lienemann, Sanna Viitanen, Pernilla Syrjä

**Affiliations:** 1 University of Helsinki, Helsinki, Finland; 2 Finnish Food Authority, Helsinki, Finland

## Clinical History, Laboratory Results, and Gross Findings

A 3-year-old, intact female border collie was referred to the University of Helsinki Veterinary Teaching Hospital in June 2023 due to coughing, fever, and increase in serum C-reactive protein concentration for 5 weeks. The referring veterinarian noted alveolar consolidation in the right middle lung lobe in thoracic radiographs. At referral, the patient was under antibiotic treatment (enrofloxacin 8 mg/kg once daily and amoxicillin and clavulanic acid 22 mg/kg 3 times a day) but not clinically improving.

The dog was febrile (39.9°C) and dehydrated when presented at the Veterinary Teaching Hospital. The dog’s capillary refill time was prolonged, indicating hypovolemia. The respiratory rate was increased (52 beats/min), mild crackles were noted in the ventral parts of the lungs during auscultation, and a productive cough was initiated by tracheal palpation.

Blood samples showed a markedly increased serum C-reactive protein concentration (132.5 mg/L; laboratory reference range: <10 mg/L), while the complete blood count was within the reference range. No significant abnormalities were noted in the serum biochemistry profile except moderate hypoalbuminemia (20.9 g/L; laboratory reference range: 30–41 g/L). An arterial sample was obtained from the metatarsal artery. Blood gas analysis revealed a moderate decrease in the PaO_2_ (70.2 mm Hg; laboratory reference range: 88–110 mm Hg). Thoracic computed tomography with intravenous iodine contrast and bronchoscopy with bronchoalveolar lavage (BAL) were performed under general anesthesia. BAL samples were processed for cytology and quantitative aerobic culture.

The right middle lung lobe was most severely affected in the thoracic computed tomography images. The entire lobe was enlarged and atelectatic with soft tissue density and rounded edges. Air filled bronchi were visible only in the proximal parts of the lobe. In addition, consolidation with soft tissue density was noted in the accessory lung lobe. Small nodules of similar appearance in thoracic computed tomography were present multifocally in other lung lobes. The tracheobronchial and mediastinal lymph nodes were mildly enlarged. Bronchoscopy revealed purulent discharge originating from the right middle lung lobe. An increased total cell count (1.12 × 10^6^/mL; laboratory reference range: 0.067–0.141 × 10^6^/mL) and neutrophilic inflammation (91.4%; laboratory reference range <7%) were noted in the BAL samples.

## Microscopic Findings

**Figure 1.**
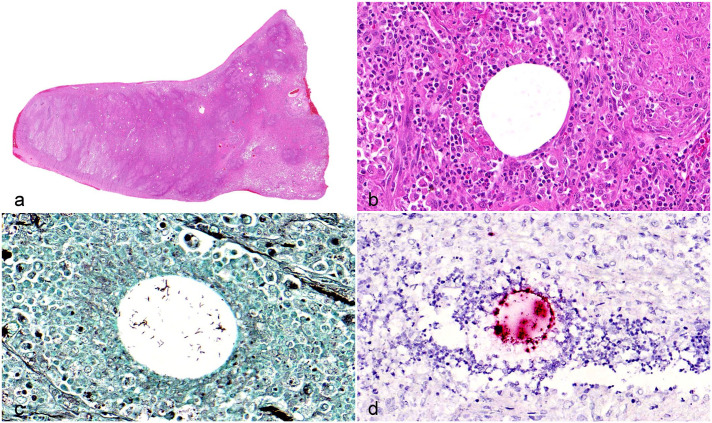
Pyogranulomatous inflammation, lung lobe, dog. (a) The lung is almost completely replaced by inflammation. Hematoxylin and eosin (HE). (b) Macrophages, lymphocytes, plasma cells, and some neutrophils are arranged around a central clear vacuole. HE. (c) Within the clear vacuole there are clusters of rod-shaped bacterial. Grocott’s methenamine silver stain. (d) Chromogenic in situ hybridization labels bacteria (red) in the clear vacuole. Chromogenic in situ hybridization for *16S rRNA*.

The affected lung lobe was surgically removed via thoracotomy. A fresh tissue sample was obtained for bacterial culture, while the residual lobe was fixed in 10% neutral-buffered formalin. The lung lobe was routinely processed and stained with hematoxylin and eosin. Overall, the lung lobe was almost completely replaced by multifocal to coalescing concentric foci of inflammatory cells (Fig. 1a). The nodules were mainly formed by numerous macrophages, some of which had an epithelioid morphology. A clear vacuole, surrounded by few neutrophils, was often the center of the concentrically arranged inflammatory cells (Fig. 1b). Numerous lymphocytes and plasma cells were admixed with the macrophages at the periphery of the inflammatory foci. The lung tissue affected by coalescing pyogranulomatous inflammation had random multifocal necrosis with diffuse pleural fibrosis. Neither Ziehl-Neelsen nor modified Ziehl-Neelsen staining, using soft carbol fuchsin as the primary dye and 5% sulfuric acid for differentiation, revealed acid-fast staining organisms within the pulmonary lesions.

The resected lung lobe was further examined histologically using a Grocott’s methenamine silver stain, which exposed elongated rod-shaped (15–20 µm long), thin (1–2 µm in diameter), regular structures in clusters within the clear vacuoles (Fig. 1c). Chromogenic in situ hybridization of the resected formalin-fixed pulmonary sample with a probe detecting bacterial *16S rRNA* (Bio-Techne, Advanced Cell Diagnostics, Dublin, Ireland) confirmed that the structures in the center of the granulomas were bacteria (Fig. 1d).

## Further Investigations and Diagnosis

Microbiological culture was performed from the BAL sample, the pleural effusion, and the lung biopsy at the Laboratory of Clinical Microbiology (YESLAB) at the Veterinary Teaching Hospital. After 1 day of incubation, the culture of the BAL sample fluid showed minimal growth of coagulase-negative staphylococci. After 6 days of incubation, a moderate growth of gray, matte bacterial colonies were observed, which were identified as *Mycobacterium thermoresistibile* with a confidence score of 1.94 using matrix-assisted laser desorption ionization time-of-flight mass spectrometry (MALDI-TOF MS, Bruker microflex LT, Bruker GmbH). At this stage, no growth was observed in the pleural effusion or lung biopsy cultures. To exclude mixed infection with tuberculous mycobacteria, the BAL, pleural effusion, and lung biopsy were further tested at the Finnish Food Authority by real-time PCR, targeting the *Mycobacterium tuberculosis* Complex (MTBC)-specific *IS6110* gene with negative results.^5^ Upon re-examination of the primary culture media after 2 weeks of cultivation at YESLAB, heavy growth of *M. thermoresistibile* was evident on both the lung biopsy and pleural effusion plates. Finally, mycobacterial culture performed from the referred samples at the Finnish Food Authority also revealed scant growth of mycobacterium species, identified as *M. thermoresistibile* with a confidence score of 2.05 using MALDI-TOF MS (Bruker Biotyper instrument using Bruker MBT Compass software v4 and Bruker mycobacteria library v2, Bruker Daltonics, Germany).

Susceptibility testing (ETEST, bioMérieux SA) on *M. thermoresistibile* was performed according to standards recommended by Clinical and Laboratory Standards Institute, and they were interpreted as recommended by Brown-Elliot and Woods for rapidly growing mycobacteria.^1^ The isolate was susceptible to all antimicrobials tested. The dog was treated with a combination of peroral clarithromycin, enrofloxacin, and doxycycline for 3 months and with clarithromycin and doxycycline for an additional 3 months. Treatment was based on susceptibility testing of the cultured *M. thermoresistibile.* The clinical signs gradually alleviated during treatment and serum C-reactive protein concentration normalized. At the end of the treatment period, the dog was clinically healthy and there were no signs of relapse 4 weeks after the discontinuation of antibiotics.

## Discussion

The genus *Mycobacterium* comprises bacteria characterized by unique cell wall structures. Their cell walls contain high concentrations of mycolic acids and lipids, which protect the bacteria from disinfectants, extreme temperatures, and pH fluctuations. This distinctive composition grants the bacteria acid-fast staining properties, making them easily distinguishable from other bacterial genera when stained with dyes like the Ziehl-Neelsen stain.^8^ Traditionally, mycobacteria are categorized into 2 groups; *Mycobacterium tuberculosis* complex and non-tuberculous mycobacteria, which can be further subdivided according to mycobacterial properties.^8^ However, recent genome sequencing and phylogenomic analyses have led to a reclassification of the genus mycobacteria.^3^ According to this updated classification, the genus *Mycobacterium* now only includes the “Tuberculosis-Similae” clade, while 4 other genera (*Mycolicibacterium*, *Mycolicibacter*, *Mycolicibacillus*, and *Mycobacteroides*) were created to replace the former classification.^3^ *M. thermoresistibile*, previously classified as an non-tuberculous mycobacteria, now belongs to the genera of *Mycolicibacterium*.^3^ However, the debate about the reclassification is still ongoing and arguments favoring the traditional nomenclature have been presented.^6^ Infections caused by *M. thermoresistibile* are rare in dogs and uncommon in cats.^2^ Penetrating trauma is assumed to be the cause of the infection in most cases.^2^ In histological examinations, *M. thermoresistibile* and related non-tuberculous mycobacteria tend to have a high affinity for lipid-rich tissues, leading to the frequent observation of degenerating adipocytes as a clear central zone within the pyogranuloma.^2^ In humans, *M. thermoresistibile* infections are rare, with only 9 documented cases, 4 of which were pulmonary infections.^9^

Mycobacterial infection is generally considered as an etiology by veterinary pathologists when diagnosing a granulomatous and/or pyogranulomatous lesion in tissue. Ziehl-Neelsen stainings are widely used standard histochemical methods for verifying mycobacteriosis in such cases. Acid-fast stains are widely available, fast diagnostic methods, which may be crucial since this bacterial genus contains several species with zoonotic potential and are comparably slow growing in culture. However, the sensitivity of acid-fast staining might not be sufficient.^4^ When mycobacteria die in tissues due to antibiotic treatment, the bacteria lose their fatty capsule and consequently their carbol fuchsin positivity, which is seen as red, in the Ziehl-Neelsen staining.^7^ In general, administration of antibiotics before a biopsy often causes false-negative results for detecting microorganisms in special stains.^7^ In this case, loss of acid-fast properties may have occurred because of longstanding antibiotics. Carbohydrates in the cell walls can still be demonstrated by Grocott’s methenamine silver stain.^7^ Grocott’s methenamine silver staining is a relatively easy, fast, and low-cost diagnostic method and, can be useful in cases where acid-fast staining procedures fail.^10^ Grocott’s methenamine silver stain might be especially useful in cases where the number of bacteria is low or hidden by inflammation.^10^

This case highlights the importance of molecular methods for detecting bacterial infection, in addition to standard histochemical stains, in cases with a history of prolonged antibiotic treatment and pyogranulomatous lesions.
